# The Adaptive Dynamic Programming Toolbox

**DOI:** 10.3390/s21165609

**Published:** 2021-08-20

**Authors:** Xiaowei Xing, Dong Eui Chang

**Affiliations:** School of Electrical Engineering, Korea Advanced Institute of Science and Technology, Daejeon 34141, Korea; xwxing@kaist.ac.kr

**Keywords:** adaptive dynamic programming, optimal control, software package

## Abstract

The paper develops the adaptive dynamic programming toolbox (ADPT), which is a MATLAB-based software package and computationally solves optimal control problems for continuous-time control-affine systems. The ADPT produces approximate optimal feedback controls by employing the adaptive dynamic programming technique and solving the Hamilton–Jacobi–Bellman equation approximately. A novel implementation method is derived to optimize the memory consumption by the ADPT throughout its execution. The ADPT supports two working modes: model-based mode and model-free mode. In the former mode, the ADPT computes optimal feedback controls provided the system dynamics. In the latter mode, optimal feedback controls are generated from the measurements of system trajectories, without the requirement of knowledge of the system model. Multiple setting options are provided in the ADPT, such that various customized circumstances can be accommodated. Compared to other popular software toolboxes for optimal control, the ADPT features computational precision and time efficiency, which is illustrated with its applications to a highly non-linear satellite attitude control problem.

## 1. Introduction

Optimal control is an important branch in control engineering. For continuous-time dynamical systems, finding an optimal feedback control involves solving the so-called Hamilton–Jacobi–Bellman (HJB) equation [1]. For linear systems, however, the HJB equation simplifies to the well-known Riccati equation which results in the linear quadratic regulator [2]. For non-linear systems, solving the HJB equation is generally a formidable task due to its inherently non-linear nature. As a result, there has been a great deal of research devoted to approximately solving the HJB equation. Al’brekht proposed a power series method for smooth systems to solve the HJB equation [3]. Under the assumption that the optimal control and the optimal cost function can be represented in Taylor series, by plugging the series expansions of the dynamics, the cost integrand function, the optimal control and the optimal cost function into the HJB equation and collecting terms degree by degree, the Taylor expansions of the optimal control and the optimal cost function can be recursively obtained. Similar ideas can be found in [4,5]. A recursive algorithm is developed to sequentially improve the control law which converges to the optimal one by starting with an admissible control [6]. This recursive algorithm is commonly referred to as policy iteration (PI) and can be also found in [7,8,9]. The common limitation of these methods is that the complete knowledge of the system is required.

In the past few decades, reinforcement learning (RL) [10] has provided a means to design optimal controllers in an adaptive manner from the viewpoint of learning. Adaptive or approximate dynamic programming (ADP), which is an iterative RL-based adaptive optimal control design method, has been proposed in [11,12,13,14,15]. An approach that employs ADP is proposed in [11] for linear systems without requiring the priori knowledge of the system matrices. An ADP strategy is presented for non-linear systems with partially unknown dynamics in [12], and the necessity of the knowledge of system model is fully relaxed in [13,14,15].

Together with the growth of optimal control theory and methods, several software tools for optimal control have been developed. Notable examples are non-linear systems toolbox [16], control toolbox [17], ACADO [18], its successor ACADOS [19], and GPOPS-II [20]. A common feature of these packages is that system equations are used in them. In addition, optimal controls generated by [17,18,19,20] are open-loop, such that an optimal control is computed for each initial state. Therefore, if the initial state changes, optimal controls need to be computed again. In contrast, the non-linear systems toolbox [16] produces an optimal feedback control by solving the HJB equation.

The primary objective of this paper is to develop a MATLAB-based toolbox that solves optimal feedback control problems computationally for control-affine systems in the continuous-time domain. More specifically, employing the adaptive dynamic programming technique, we derive a computational methodology to compute approximate optimal feedback controls, based on which we develop the adaptive dynamic programming toolbox (ADPT). In the derivation, the Kronecker product used in [11,14] is replaced by Euclidean inner product for the purpose of memory saving during execution of the ADPT. The ADPT supports two working modes: the model-based mode and the model-free mode. The knowledge of system equations is required in the model-based mode. In the model-free mode, the ADPT produces the approximate optimal feedback control from measurements of system trajectories, removing the requirement of the knowledge of system equations. Moreover, multiple options are provided, such that the user can use the toolbox with much flexibility.

The remainder of the paper is organized as follows. Section 2 reviews the standard optimal control problem for a class of continuous-time non-linear systems and the model-free adaptive dynamic programming technique. Section 3 provides implementation details and software features of the ADPT. In Section 4, the ADPT is applied to a satellite attitude control problem in both the model-based mode and the model-free mode. Conclusions and potential future directions are given in Section 5. The codes of the ADPT are available at https://github.com/Everglow0214/The_Adaptive_Dynamic_Programming_Toolbox, accessed on 10 August 2021.

## 2. Review of Adaptive Dynamic Programming

We review the adaptive dynamic programming (ADP) technique to solve optimal control problems [13,14]. Consider a continuous-time control-affine system given by

(1) $$\dot{x}=f\left(x\right)+g\left(x\right)u,$$

where $x\in R^{n}$ is the state, $u\in R^{m}$ is the control, $f:R^{n}\to R^{n}$ and $g:R^{n}\to R^{n\times m}$ are locally Lipschitz mappings with f(0)=0. It is assumed that (1) is stabilizable at x=0 in the sense that the system can be locally asymptotically stabilized by a continuous feedback control. To quantify the performance of a control, an integral cost associated with (1) is given by

(2) $$J\left(x_{0},u\right)=\int_{0}^{\infty }\left(q\left(x\left(t\right)\right)+u{\left(t\right)}^{T}Ru\left(t\right)\right)\;dt,$$

where $x_{0}=x\left(0\right)$ is the initial state, $q:R^{n}\to R_{\geq 0}$ is a positive definite function and $R\in R^{m\times m}$ is a symmetric, positive definite matrix. A feedback control $u:R^{n}\to R^{m}$ is said to be admissible if it stabilizes (1) at the origin, and makes the cost $J(x_{0},u)$ finite for all $x_{0}$ in a neighborhood of x=0.

The objective is to find a control policy *u* that minimizes $J(x_{0},u)$ given $x_{0}$. Define the optimal cost function $V^{*}:R^{n}\to R$ by

$$V^{*}\left(x\right)=\min_{u}J\left(x,u\right)$$

for $x\in R^{n}$. Then, $V^{*}$ satisfies the HJB equation

$$0=\min_{u}\left\{\nabla V^{*}{\left(x\right)}^{T}\left(f\left(x\right)+g\left(x\right)u\right)+q\left(x\right)+u^{T}Ru\right\},$$

and the minimizer in the HJB equation is the optimal control which is expressed in terms of $V^{*}$ as

$$u^{*}\left(x\right)=-\frac{1}{2}R^{-1}g{\left(x\right)}^{T}\nabla V^{*}\left(x\right).$$

Moreover, the state feedback $u^{*}$ locally asymptotically stabilizes (1) at the origin and minimizes (2) over all admissible controls [2]. Solving the HJB equation analytically is extremely difficult in general except for linear cases. Hence, approximate or iterative methods are needed to solve the HJB, and the well-known policy iteration (PI) technique [6] is reviewed in Algorithm 1. Let ${\left\{V_{i}\left(x\right)\right\}}_{i\geq 0}$ and ${\left\{u_{i+1}\left(x\right)\right\}}_{i\geq 0}$ be the sequences of functions generated by PI in Algorithm 1. It is shown in [6] that $V_{i+1}\left(x\right)\leq V_{i}\left(x\right)$ for i≥0, and the limit functions $V\left(x\right)=\lim_{i\to \infty }V_{i}\left(x\right)$ and $u\left(x\right)=\lim_{i\to \infty }u_{i}\left(x\right)$ are equal to the optimal cost function $V^{*}$ and the optimal control $u^{*}$.
**Algorithm 1.** Policy iteration**Input:**  An initial admissible control $u_{0}\left(x\right)$, and a threshold ϵ>0.**Output:**  The approximate optimal control $u_{i+1}\left(x\right)$ and the approximate optimal cost function $V_{i}\left(x\right)$.1: Set i←0.2: **while** 
i≥0
**do**3:   Policy evaluation: solve for the continuously differentiable cost function $V_{i}\left(x\right)$ with     
$V_{i}\left(0\right)=0$ using
(3)$\nabla V_{i}{\left(x\right)}^{T}\left(f\left(x\right)+g\left(x\right)u_{i}\left(x\right)\right)+q\left(x\right)+u_{i}{\left(x\right)}^{T}Ru_{i}\left(x\right)=0.$4:   Policy improvement: update the control policy by
(4)$$u_{i+1}\left(x\right)=-\frac{1}{2}R^{-1}g{\left(x\right)}^{T}\nabla V_{i}\left(x\right).$$5:   **if** $∥u_{i+1}\left(x\right)-u_{i}\left(x\right)∥\leq \epsilon$ for all *x*
**then**6:       **break**7:   **end if**8:   Set i←i+1.9: **end while**

As proposed in [13,14], consider approximating the solutions to (3) and (4) by ADP instead of obtaining them exactly. For this purpose, choose an admissible feedback control $u_{0}:R^{n}\to R^{m}$ for (1) and let ${\left\{V_{i}\left(x\right)\right\}}_{i\geq 0}$ and ${\left\{u_{i+1}\left(x\right)\right\}}_{i\geq 0}$ be the sequences of functions generated by PI in Algorithm 1 starting with the control $u_{0}\left(x\right)$. Following [13,14], choose a bounded time-varying exploration signal $\eta :R\to R^{m}$, and apply the sum $u_{0}\left(x\right)+\eta \left(t\right)$ to (1) as follows:

(5) $$\dot{x}=f\left(x\right)+g\left(x\right)\left(u_{0}\left(x\right)+\eta \left(t\right)\right).$$

Assume that solutions to (5) are well defined for all positive time. Let $T\left(x,u_{0},\eta ,\left[r,s\right]\right)=\left\{\left(x\left(t\right),u_{0}\left(x\left(t\right)\right),\eta \left(t\right)\right)|r\leq t\leq s\right\}$ denote the trajectory x(t) of the system (5) with the input $u_{0}+\eta$ over the time interval [r,s] with 0≤r<s. The system (5) can be rewritten as

(6) $$\dot{x}=f\left(x\right)+g\left(x\right)u_{i}\left(x\right)+g\left(x\right)\nu_{i}\left(x,t\right),$$

where

$$\nu_{i}\left(x,t\right)=u_{0}\left(x\right)-u_{i}\left(x\right)+\eta \left(t\right).$$

Combined with (3) and (4), the time derivative of $V_{i}\left(x\right)$ along the trajectory x(t) of (6) is obtained as

(7) $${\dot{V}}_{i}\left(x\right)=-q\left(x\right)-u_{i}{\left(x\right)}^{T}Ru_{i}\left(x\right)-2u_{i+1}{\left(x\right)}^{T}R\nu_{i}\left(x,t\right)$$

for i≥0. By integrating both sides of (7) over any time interval [r,s] with 0≤r<s, one gets

(8) $$V_{i}\left(x\left(s\right)\right)-V_{i}\left(x\left(r\right)\right)=-\int_{r}^{s}\left(q\left(x\right)+u_{i}{\left(x\right)}^{T}Ru_{i}\left(x\right)+2u_{i+1}{\left(x\right)}^{T}R\nu_{i}\left(x,\tau \right)\right)\;d\tau .$$

Let $\phi_{j}:R^{n}\to R$ and $\varphi_{j}:R^{n}\to R^{m}$, with j=1,2,…, be two infinite sequences of continuous basis functions on a compact set in $R^{n}$ containing the origin as an interior point that vanish at the origin [13,14]. Then, $V_{i}\left(x\right)$ and $u_{i+1}\left(x\right)$ for each i≥0 can be expressed as infinite series of the basis functions. For each i≥0 let ${\hat{V}}_{i}\left(x\right)$ and ${\hat{u}}_{i+1}\left(x\right)$ be approximations of $V_{i}\left(x\right)$ and $u_{i+1}\left(x\right)$ given by

(9) $$\begin{array}{cc} {\hat{V}}_{i}\left(x\right) & =\sum_{j=1}^{N_{1}}c_{i,j}\phi_{j}\left(x\right), \end{array}$$

(10) $$\begin{array}{cc} {\hat{u}}_{i+1}\left(x\right) & =\sum_{j=1}^{N_{2}}w_{i,j}\varphi_{j}\left(x\right), \end{array}$$

where $N_{1}>0$ and $N_{2}>0$ are integers and $c_{i,j}$, $w_{i,j}\in R$ are coefficients to be found for each i≥0. Then, Equation (8) is approximated by ${\hat{V}}_{i}\left(x\right)$ and ${\hat{u}}_{i+1}\left(x\right)$ as follows:

(11) $$\begin{array}{cc} \; & \sum_{j=1}^{N_{1}}c_{i,j}\left(\phi_{j}\left(x\left(s\right)\right)-\phi_{j}\left(x\left(r\right)\right)\right)+\int_{r}^{s}\left(2\sum_{j=1}^{N_{2}}w_{i,j}\varphi_{j}{\left(x\right)}^{T}R{\hat{\nu }}_{i}\right)\;d\tau \\ \; & \;\;=-\int_{r}^{s}\left(q\left(x\right)+{\hat{u}}_{i}{\left(x\right)}^{T}R{\hat{u}}_{i}\left(x\right)\right)\;d\tau , \end{array}$$

where

(12) $${\hat{u}}_{0}=u_{0},\;{\hat{\nu }}_{i}=u_{0}-{\hat{u}}_{i}+\eta .$$

Suppose that we have *K* trajectories $T(x,u_{0},\eta ,\left[r_{k},s_{k}\right])$ available, k=1,…,K, where x(t), $u_{0}\left(t\right)$, and η(t) satisfy (6) over the *K* time intervals $\left[r_{k},s_{k}\right]$, k=1,…,K. Then, we have *K* equations of the form (11) for each i≥0, which can be written as

(13) $$e_{i,k}=0,\;k=1,\ldots ,K,$$

where

$$\begin{array}{cc} e_{i,k}:= & \sum_{j=1}^{N_{1}}c_{i,j}\left(\phi_{j}\left(x\left(s_{k}\right)\right)-\phi_{j}\left(x\left(r_{k}\right)\right)\right)+\int_{r_{k}}^{s_{k}}\left(2\sum_{j=1}^{N_{2}}w_{i,j}\varphi_{j}{\left(x\right)}^{T}R{\hat{\nu }}_{i}\right)\;d\tau \\ \; & +\int_{r_{k}}^{s_{k}}\left(q\left(x\right)+{\hat{u}}_{i}{\left(x\right)}^{T}R{\hat{u}}_{i}\left(x\right)\right)\;d\tau . \end{array}$$

Then, the coefficients ${\left\{c_{i,j}\right\}}_{j=1}^{N_{1}}$ and ${\left\{w_{i,j}\right\}}_{j=1}^{N_{2}}$ are obtained by minimizing

$$\sum_{k=1}^{K}{∥e_{i,k}∥}^{2}.$$

In other words, the *K* equations in (13) are solved in the least squares sense for the coefficients, ${\left\{c_{i,j}\right\}}_{j=1}^{N_{1}}$ and ${\left\{w_{i,j}\right\}}_{j=1}^{N_{2}}$. Thus two sequences ${\left\{{\hat{V}}_{i}\left(x\right)\right\}}_{i=0}^{\infty }$ and ${\left\{{\hat{u}}_{i+1}\left(x\right)\right\}}_{i=0}^{\infty }$ can be generated from (11). According to ([14], Cor. 3.2.4), for any arbitrary ϵ>0, there exist integers $i^{*}>0$, $N_{1}^{**}>0$ and $N_{2}^{**}>0$, such that

$$\begin{array}{cc} ∥\sum_{j=1}^{N_{1}}c_{i^{*},j}\phi_{j}\left(x\right)-V^{*}\left(x\right)∥ & \leq \epsilon ,\\ ∥\sum_{j=1}^{N_{2}}w_{i^{*},j}\varphi_{j}\left(x\right)-u^{*}\left(x\right)∥ & \leq \epsilon \end{array}$$

for all *x* in a neighborhood of the origin, if $N_{1}>N_{1}^{**}$ and $N_{2}>N_{2}^{**}$.

**Remark** **1.** *The ADP algorithm relies only on the measurements of states, the initial control policy and the exploration signal, lifting the requirement of knowing the precise system model, while the conventional policy iteration algorithm in Algorithm 1 requires the knowledge of the exact system model. Hence, the ADP algorithm is 100% data-based and model-free.*

**Remark** **2.** *Equation (11) depends on the initial control $u_{0}$, the exploration signal η, the time interval [r,s] as well as the index i, where the first three $u_{0}$, η, and [r,s] are together equivalent to the trajectory $T(x,u_{0},\eta ,\left[r,s\right])$ if the initial state x(r) at t=r is given. Hence, we can generate more diverse trajectories by changing η and [r,s], as well as the initial state, and enrich the ADP algorithm accordingly, as follows. Suppose that we have available K trajectories $T(x^{k},u_{0},\eta^{k},\left[r_{k},s_{k}\right])$, 1≤k≤K, where $x^{k}$, $u_{0}$ and $\eta^{k}$ satisfy (6), i.e.,*

$${\dot{x}}^{k}\left(t\right)=f\left(x^{k}\left(t\right)\right)+g\left(x^{k}\left(t\right)\right)\left(u_{0}\left(x^{k}\left(t\right)\right)+\eta^{k}\left(t\right)\right)$$

*for $r_{k}\leq t\leq s_{k}$. Then, we have K equations of the form (11) for each i≥0, which can be written as $e_{i,k}=0$, k=1,…,K, where*

$$\begin{array}{cc} e_{i,k}:= & \sum_{j=1}^{N_{1}}c_{i,j}\left(\phi_{j}\left(x^{k}\left(s_{k}\right)\right)-\phi_{j}\left(x^{k}\left(r_{k}\right)\right)\right)+\int_{r_{k}}^{s_{k}}\left(2\sum_{j=1}^{N_{2}}w_{i,j}\varphi_{j}{\left(x^{k}\right)}^{T}R{\hat{\nu }}_{i}^{k}\right)\;d\tau \\ \; & +\int_{r_{k}}^{s_{k}}\left(q\left(x^{k}\right)+{\hat{u}}_{i}{\left(x^{k}\right)}^{T}R{\hat{u}}_{i}\left(x^{k}\right)\right)\;d\tau \end{array}$$

*with ${\hat{u}}_{0}=u_{0}$ and ${\hat{\nu }}_{i}^{k}=u_{0}+\eta^{k}-{\hat{u}}_{i}$. Then, the coefficients ${\left\{c_{i,j}\right\}}_{j=1}^{N_{1}}$ and ${\left\{w_{i,j}\right\}}_{j=1}^{N_{2}}$ are obtained by minimizing $\sum_{k=1}^{K}{∥e_{i,k}∥}^{2}$. For the sake of simplicity of presentation, however, in this paper we will fix η and the initial states and vary only the time intervals to generate trajectory data.*

## 3. Implementation Details and Software Features

We now discuss implementation details and features of the adaptive dynamic programming toolbox (ADPT). We provide two modes to generate approximate optimal feedback controls; one mode requires the knowledge of system model, but the other eliminates this requirement, giving rise to the ADPT’s unique capability of handling model-free cases.

### 3.1. Implementation of Computational Adaptive Dynamic Programming

To approximate $V_{i}\left(x\right)$ and $u_{i+1}\left(x\right)$ in (3) and (4), monomials composed of state variables are selected as basis functions. For a pre-fixed number d≥1, define a column vector $\Phi_{d}\left(x\right)$ by ordering monomials in graded reverse lexicographic order [21] as

$$\Phi_{d}\left(x\right)=\left(x_{1},\ldots ,x_{n},x_{1}^{2},x_{1}x_{2},\ldots ,x_{n}^{2},\ldots ,x_{n}^{d}\right)\in R^{N\times 1},$$

where $x=\left(x_{1},x_{2},\ldots ,x_{n}\right)\in R^{n}$ is the state, d≥1 is the highest degree of the monomials, and *N* is given by

N=∑i=1di+n−1n−1.

For example, if n=3 and d=3, the corresponding ordered monomials are

$$\begin{array}{cc} \; & x_{1},x_{2},x_{3};\\ \; & x_{1}^{2},x_{1}x_{2},x_{1}x_{3},x_{2}^{2},x_{2}x_{3},x_{3}^{2};\\ \; & x_{1}^{3},x_{1}^{2}x_{2},x_{1}^{2}x_{3},x_{1}x_{2}^{2},x_{1}x_{2}x_{3},x_{1}x_{3}^{2},x_{2}^{3},x_{2}^{2}x_{3},x_{2}x_{3}^{2},x_{3}^{3}. \end{array}$$

According to (9) and (10), the cost function $V_{i}\left(x\right)$ and the control $u_{i+1}\left(x\right)$ are approximated by ${\hat{V}}_{i}\left(x\right)$ and ${\hat{u}}_{i+1}\left(x\right)$, which are defined as

(14) $$\begin{array}{cc} {\hat{V}}_{i}\left(x\right) & =c_{i}\Phi_{d+1}\left(x\right), \end{array}$$

(15) $$\begin{array}{cc} {\hat{u}}_{i+1}\left(x\right) & =W_{i}\Phi_{d}\left(x\right), \end{array}$$

where d≥1 is the approximation degree, and $c_{i}\in R^{1\times N_{1}}$ and $W_{i}\in R^{m\times N_{2}}$ are composed of coefficients corresponding to the monomials in $\Phi_{d+1}\left(x\right)$ and $\Phi_{d}\left(x\right)$ with

N1=∑i=1d+1i+n−1n−1,N2=∑i=1di+n−1n−1.

We take the highest degree of monomials to approximate $V_{i}$ greater by one than the approximation degree since $u_{i+1}$ is obtained by taking the gradient of $V_{i}$ in (4) and g(x) is constant in most cases.

**Theorem** **1.** *Let a set of trajectories be defined as $S_{T}=\left\{T\left(x,u_{0},\eta ,\left[r_{k},s_{k}\right]\right),k=1,2,\ldots ,K\right\}$ with K≥1, and let*

$$\begin{array}{cc} \alpha (x) & =R\eta \Phi_{d}{\left(x\right)}^{T},\\ \beta (x) & =R\left(u_{0}\left(x\right)+\eta \right)\Phi_{d}{\left(x\right)}^{T},\\ \gamma (x) & =\Phi_{d}\left(x\right)\Phi_{d}{\left(x\right)}^{T}. \end{array}$$

*Then the coefficients $c_{i}$ and $W_{i}$ satisfy*

(16) $$A_{i}\left[\begin{array}{c} c_{i}^{T}\\ \mathrm{vec}(W_{i}) \end{array}\right]=b_{i},$$

*where*

$$\begin{array}{cc} A_{0} & =\left[\begin{array}{cc} \Phi_{d+1}^{\left[r_{1},s_{1}\right]}\left(x\right) & 2\mathrm{vec}{\left(\int_{r_{1}}^{s_{1}}\alpha \left(x\right)\;dt\right)}^{T}\\ \vdots & \vdots \\ \Phi_{d+1}^{\left[r_{K},s_{K}\right]}\left(x\right) & 2\mathrm{vec}{\left(\int_{r_{K}}^{s_{K}}\alpha \left(x\right)\;dt\right)}^{T} \end{array}\right]\in R^{K\times (N_{1}+mN_{2})},\\ b_{0} & =\left[\begin{array}{c} -\int_{r_{1}}^{s_{1}}\left(q\left(x\right)+u_{0}{\left(x\right)}^{T}Ru_{0}\left(x\right)\right)\;dt\\ \vdots \\ -\int_{r_{K}}^{s_{K}}\left(q\left(x\right)+u_{0}{\left(x\right)}^{T}Ru_{0}\left(x\right)\right)\;dt \end{array}\right]\in R^{K\times 1}, \end{array}$$

*and for i=1,2,…,*

$$\begin{array}{cc} A_{i} & =\left[\begin{array}{cc} \Phi_{d+1}^{\left[r_{1},s_{1}\right]}\left(x\right) & 2\mathrm{vec}{\left(\int_{r_{1}}^{s_{1}}\left(\beta \left(x\right)-RW_{i-1}\gamma \left(x\right)\right)\;dt\right)}^{T}\\ \vdots & \vdots \\ \Phi_{d+1}^{\left[r_{K},s_{K}\right]}\left(x\right) & 2\mathrm{vec}{\left(\int_{r_{K}}^{s_{K}}\left(\beta \left(x\right)-RW_{i-1}\gamma \left(x\right)\right)\;dt\right)}^{T} \end{array}\right]\in R^{K\times (N_{1}+mN_{2})},\\ b_{i} & =\left[\begin{array}{c} -\int_{r_{1}}^{s_{1}}q\left(x\right)\;dt-\langle W_{i-1}^{T}RW_{i-1},\int_{r_{1}}^{s_{1}}\gamma \left(x\right)\;dt\rangle \\ \vdots \\ -\int_{r_{K}}^{s_{K}}q\left(x\right)\;dt-\langle W_{i-1}^{T}RW_{i-1},\int_{r_{K}}^{s_{K}}\gamma \left(x\right)\;dt\rangle \end{array}\right]\in R^{K\times 1}, \end{array}$$

*where*

$$\begin{array}{c} \Phi_{d+1}^{\left[r_{k},s_{k}\right]}\left(x\right)=\Phi_{d+1}{\left(x\left(s_{k}\right)\right)}^{T}-\Phi_{d+1}{\left(x\left(r_{k}\right)\right)}^{T} \end{array}$$

*for k=1,2,…,K, the operator 〈·,·〉 denotes the Euclidean inner product with $\langle E,F\rangle =\sum_{ij}E_{ij}F_{ij}$ for matrices $E=[E_{ij}]$ and $F=[F_{ij}]$ of equal size, and the operator vec(·) is defined as*

$$\begin{array}{c} \mathrm{vec}\left(Z\right)=\left[\begin{array}{c} z_{1}\\ z_{2}\\ \vdots \\ z_{n} \end{array}\right]\in R^{mn\times 1} \end{array}$$

*with $z_{j}\in R^{m\times 1}$ being the jth column of a matrix $Z\in R^{m\times n}$ for j=1,…,n.*

**Proof.**  Combining (11), (14) and (15), one has

(17) $$\begin{array}{cc} \; & c_{0}\left(\Phi_{d+1}\left(x\left(s_{k}\right)\right)-\Phi_{d+1}\left(x\left(r_{k}\right)\right)\right)+2\int_{r_{k}}^{s_{k}}\Phi_{d}{\left(x\right)}^{T}W_{0}^{T}R\eta \;dt\\ \; & \;\;=-\int_{r_{k}}^{s_{k}}\left(q\left(x\right)+u_{0}{\left(x\right)}^{T}Ru_{0}\left(x\right)\right)\;dt, \end{array}$$

and for i=1,2,…,

(18) $$\begin{array}{cc} \; & c_{i}\left(\Phi_{d+1}\left(x\left(s_{k}\right)\right)-\Phi_{d+1}\left(x\left(r_{k}\right)\right)\right)+2\int_{r_{k}}^{s_{k}}\Phi_{d}{\left(x\right)}^{T}W_{i}^{T}R\left(u_{0}\left(x\right)+\eta \right)\;dt\\ \; & -2\int_{r_{k}}^{s_{k}}\Phi_{d}{\left(x\right)}^{T}W_{i}^{T}RW_{i-1}\Phi_{d}\left(x\right)\;dt\\ \; & \;=-\int_{r_{k}}^{s_{k}}\left(q\left(x\right)+\Phi_{d}{\left(x\right)}^{T}W_{i-1}^{T}RW_{i-1}\Phi_{d}\left(x\right)\right)\;dt. \end{array}$$

By applying the property

$$\langle A,BC\rangle =\langle AC^{T},B\rangle =\langle B^{T}A,C\rangle$$

of the Euclidean inner product, one may rewrite (17) and (18) as

(19) $$\begin{array}{cc} \; & c_{0}\left(\Phi_{d+1}\left(x\left(s_{k}\right)\right)-\Phi_{d+1}\left(x\left(r_{k}\right)\right)\right)+2\langle W_{0},\;\int_{r_{k}}^{s_{k}}R\eta \Phi_{d}{\left(x\right)}^{T}\;dt\rangle \\ \; & \;\;=-\int_{r_{k}}^{s_{k}}\left(q\left(x\right)+u_{0}{\left(x\right)}^{T}Ru_{0}\left(x\right)\right)\;dt, \end{array}$$

and for i=1,2,…,

(20) $$\begin{array}{cc} \; & c_{i}\left(\Phi_{d+1}\left(x\left(s_{k}\right)\right)-\Phi_{d+1}\left(x\left(r_{k}\right)\right)\right)+2\langle W_{i},\;\int_{r_{k}}^{s_{k}}R\left(u_{0}\left(x\right)+\eta \right)\Phi_{d}{\left(x\right)}^{T}\;dt\rangle \\ \; & -2\langle W_{i},\;RW_{i-1}\int_{r_{k}}^{s_{k}}\Phi_{d}\left(x\right)\Phi_{d}{\left(x\right)}^{T}\;dt\rangle \\ \; & \;=-\langle W_{i-1}^{T}RW_{i-1},\;\int_{r_{k}}^{s_{k}}\Phi_{d}\left(x\right)\Phi_{d}{\left(x\right)}^{T}\;dt\rangle -\int_{r_{k}}^{s_{k}}q\left(x\right)\;dt. \end{array}$$

Then, the system of linear equations in (16) readily follows from (19) and (20).    □

We now give the computational adaptive dynamic programming algorithm in Algorithm 2 for practical implementation. To solve the least squares problem in line 5 in the algorithm, we need to have a sufficiently large number *K* of trajectories, such that the minimization problem can be solved well numerically. Then the approximate optimal feedback control is generated by the algorithm as ${\hat{u}}_{i+1}=W_{i}\Phi_{d}\left(x\right)$.
**Algorithm 2.** Computational adaptive dynamic programming**Input:** An approximation degree d≥1, an initial admissible control $u_{0}\left(x\right)$, an exploration signal η(t), and a threshold ϵ>0.**Output:** The approximate optimal control ${\hat{u}}_{i+1}\left(x\right)$ and the approximate optimal cost function ${\hat{V}}_{i}\left(x\right)$.  1:  Apply $u=u_{0}+\eta$ as the input during a sufficiently long period and collect necessary data.  2:  Set i←0.  3:  **while** 
i≥0 
**do**  4:  Generate $A_{i}$ and $b_{i}$.  5:  Obtain $c_{i}$ and $W_{i}$ by solving the minimization problem
$$\min_{c_{i},W_{i}}{∥A_{i}\left[\begin{array}{c} c_{i}^{T}\\ \mathrm{vec}\left(W_{i}\right) \end{array}\right]-b_{i}∥}^{2}.$$  6:  **if** i≥1 and ${∥c_{i}-c_{i-1}∥}^{2}+{∥W_{i}-W_{i-1}∥}^{2}\leq \epsilon^{2}$ **then**  7:        **break**  8:  **end if**  9:  Set i←i+1.10:  **end while**11:  **return** ${\hat{u}}_{i+1}\left(x\right)=W_{i}\Phi_{d}\left(x\right)$ and ${\hat{V}}_{i}\left(x\right)=c_{i}\Phi_{d+1}\left(x\right)$

**Remark** **3.** *As in the statement of Theorem 1, several integral terms are included in $A_{i}$ and $b_{i}$ for i≥0. As in (12), $u_{0}$ does not get approximated by the basis functions, so the matrices $A_{0}$ and $b_{0}$ in Theorem 1 are obtained with $x(r_{k})$, $x(s_{k})$, $\int_{r_{k}}^{s_{k}}q\left(x\right)\;dt$, $\int_{r_{k}}^{s_{k}}u_{0}{\left(x\right)}^{T}Ru_{0}\left(x\right)\;dt$ and $\int_{r_{k}}^{s_{k}}\alpha \left(x\right)\;dt$, 1≤k≤K. For i≥1, the matrices $A_{i}$ and $b_{i}$ in Theorem 1 need, in addition, $\int_{r_{k}}^{s_{k}}\beta \left(x\right)\;dt$ and $\int_{r_{k}}^{s_{k}}\gamma \left(x\right)\;dt$, 1≤k≤K, as well as $W_{i-1}$.*

**Remark** **4.** *In Theorem 1, the Kronecker product that is used in [11,14] for practical implementation is replaced by Euclidean inner product. Notice that $\int_{r_{k}}^{s_{k}}\gamma \left(x\right)\;dt\in R^{N_{2}\times N_{2}}$ is symmetric, k=1,…,K. Thus, only upper triangular elements of these matrices are required to be stored. On the other hand, by using Kronecker product, one has to save all the elements of these matrices. As a result, less memory space of the processor is occupied by Theorem 1 especially when the number of basis functions to represent the approximate optimal control is large.*

**Remark** **5.** *In the situation where the system dynamic equations are known, the ADPT uses the Runge–Kutta method to simultaneously compute the trajectory points $x(r_{k})$ and $x(s_{k})$ and the integral terms that appear in $A_{i}$ and $b_{i}$. In the case when system equations are not known but trajectory data are available, the ADPT applies the trapezoidal method to evaluate these integrals numerically. In this case, each trajectory $T(x,u_{0},\eta ,\left[r_{k},s_{k}\right])$ is represented by a set of its sample points ${\left\{x\left(t_{k,\ell }\right),u_{0}\left(t_{k,\ell }\right),\eta \left(t_{k,\ell }\right)\right\}}_{\ell =1}^{L_{k}}$, where ${\left\{t_{k,\ell }\right\}}_{\ell =1}^{L_{k}}$ is a finite sequence that satisfies $r_{k}=t_{k,1}<t_{k,2}<\ldots <t_{k,L_{k}-1}<t_{k,L_{k}}=s_{k}$, and then the trapezoidal method is applied on these sample points to numerically evaluate the integrals over the time interval $\left[r_{k},s_{k}\right]$. If intermediate points in the interval $\left[r_{k},s_{k}\right]$ are not available so that partitioning the interval $\left[r_{k},s_{k}\right]$ is impossible, then we use the two end points $r_{k}$ and $s_{k}$ to evaluate the integral by the trapezoidal method as*

(21) $$\int_{r_{k}}^{s_{k}}h\left(t\right)\;dt\approx \frac{\left(s_{k}-r_{k}\right)\left(h\left(s_{k}\right)+h\left(r_{k}\right)\right)}{2}$$

*for a function h(t).*

### 3.2. Software Features

The codes of the ADPT are available at https://github.com/Everglow0214/The_Adaptive_Dynamic_Programming_Toolbox, accessed on 10 August 2021.

#### 3.2.1. Symbolic Expressions

It is of great importance for an optimal control package that the user can describe functions, such as system equations, cost functions, etc., in a convenient manner. The idea of the ADPT is to use symbolic expressions. Consider an optimal control problem, where the system model is in the form (1) with

(22) $$\begin{array}{c} f\left(x\right)=\left[\begin{array}{c} x_{2}\\ \frac{-k_{1}x_{1}-k_{2}x_{1}^{3}-k_{3}x_{2}}{k_{4}} \end{array}\right],\;g\left(x\right)=\left[\begin{array}{c} 0\\ \frac{1}{k_{4}} \end{array}\right], \end{array}$$

where $x=\left(x_{1},x_{2}\right)\in R^{2}$ is the state, u∈R is the control, and $k_{1},k_{2},k_{3},k_{4}\in R$ are system parameters. The cost function is in the form (2) with

(23) $$\begin{array}{c} q\left(x\right)=5x_{1}^{2}+3x_{2}^{2},\;R=2. \end{array}$$

Then in the ADPT the system dynamics and the cost function can be defined in lines 1–17 in Listing A1 provided in the Appendix A.

#### 3.2.2. Working Modes

Two working modes are provided in the ADPT; the model-based mode and the model-free mode. The model-based mode deals with the situation where the system model is given, while the model-free mode addresses the situation where the system model is not known but only trajectory data are available. An example of the model-based mode is given in Listing A1, where after defining the system model (22), the cost function (23) and the approximation degree *d* in lines 1–20, the function, adpModelBased, returns the coefficients $W_{i}$ and $c_{i}$ for the control ${\hat{u}}_{i+1}$ and the cost function ${\hat{V}}_{i}$, respectively, in line 21.

An example of the model-free mode is shown in Listing A2 in the Appendix A, where the system model (22) is assumed to be unknown. The initial control $u_{0}$ is in the form of $u_{0}\left(x\right)=-Fx$ with the feedback control gain *F* defined in line 18. The exploration signal η is composed of four sinusoidal signals, as shown in lines 21–22. A list of two initial states x(0)=(−3,2) and x(0)=(2.2,3) is given in lines 28–29, and a list of the corresponding total time span for simulation is given in lines 30–31, where the time interval [0,6] is divided into sub-intervals of size 0.002 so that trajectory data are recorded every 0.002 second in lines 36–41. The time stamps are saved in the column vector t_save in line 39, and the values of states are saved in the matrix x_save in line 40, with each row in x_save corresponding to the same row in t_save. Similarly, the values of the initial control $u_{0}$ and the exploration signal η are saved in vectors u0_save and eta_save in lines 43–44. These measurements are passed to the function, adpModelFree, in lines 48–49 to compute the optimal control and the optimal cost function approximately.

In both the model-based and model-free modes the approximate control is saved in the file, uAdp.m, that is generated automatically and can be applied by calling u=uAdp(x) without dependence on other files. Similarly, the user may also check the approximate cost through the file, VAdp.m.

#### 3.2.3. Options

Multiple options are provided such that the user may customize optimal control problems in a convenient way. We here illustrate usage of some of the options, referring the reader for the other options to the user manual available at https://github.com/Everglow0214/The_Adaptive_Dynamic_Programming_Toolbox, accessed on 10 August 2021.

In the model-based mode, the user may set option values through the function, adpSetModelBased, in a name-value manner before calling adpModelBased. That is, the specified values may be assigned to the named options. An example is shown in Listing A3 in the Appendix A, where two sets of initial states, time intervals and exploration signals are specified in lines 1–9. Then, in line 15 the output of adpSetModelBased should be passed to adpModelBased for the options to take effect. Otherwise, the default values would be used for the options as in line 21 in Listing A1.

For the command, adpModelFree, option values can be modified with the function, adpSetModelFree, in the name-value manner. Among the options, ‘stride’ enables the user to record values of states, initial controls and exploration signals in a high frequency for a long time, while using only a portion of them in the iteration process inside adpModelFree. To illustrate it, let each trajectory in the set $S_{T}$ of trajectories in the statement of Theorem 1 be represented by two sample points at time $r_{k}$ and $s_{k}$, that is, the trapezoidal method evaluates integrals over $\left[r_{k},s_{k}\right]$ by taking values at $r_{k}$ and $s_{k}$ as in (21). Suppose that trajectories in $S_{T}$ are consecutive, that is, $s_{k}=r_{k+1}$ for k=1,2,…,K−1. By setting ‘stride’ to a positive integer δ, the data used to generate $A_{i}$ and $b_{i}$ in Algorithm 2 become $\left\{T\left(x,u_{0},\eta ,\left[r_{1+i\delta },s_{(i+1)\delta }\right]\right),i\in N,\left(i+1\right)\delta \leq K\right\}$. For example, consider 3 consecutive trajectories $T(x,u_{0},\eta ,\left[r_{k},r_{k+1}\right])$ with k=1,2,3. If ‘stride’ is set to 1, one will have three equations from (11) as follows:

$$\begin{array}{cc} \; & \sum_{j=1}^{N_{1}}c_{i,j}\left(\phi_{j}\left(x\left(r_{k+1}\right)\right)-\phi_{j}\left(x\left(r_{k}\right)\right)\right)+\int_{r_{k}}^{r_{k+1}}\left(2\sum_{j=1}^{N_{2}}w_{i,j}\varphi_{j}{\left(x\right)}^{T}R{\hat{\nu }}_{i}\right)\;d\tau \\ \; & \;=-\int_{r_{k}}^{r_{k+1}}\left(q\left(x\right)+{\hat{u}}_{i}{\left(x\right)}^{T}R{\hat{u}}_{i}\left(x\right)\right)\;d\tau \end{array}$$

for k=1,2,3. These three equations contribute to three rows of $A_{i}$ and three rows of $b_{i}$ as in Theorem 1. If ‘stride’ is set to 3, then one will have only one equation from (11) as follows:

(24) $$\begin{array}{cc} \; & \sum_{j=1}^{N_{1}}c_{i,j}\left(\phi_{j}\left(x\left(r_{4}\right)\right)-\phi_{j}\left(x\left(r_{1}\right)\right)\right)+\int_{r_{1}}^{r_{4}}\left(2\sum_{j=1}^{N_{2}}w_{i,j}\varphi_{j}{\left(x\right)}^{T}R{\hat{\nu }}_{i}\right)\;d\tau \\ \; & \;=-\int_{r_{1}}^{r_{4}}\left(q\left(x\right)+{\hat{u}}_{i}{\left(x\right)}^{T}R{\hat{u}}_{i}\left(x\right)\right)\;d\tau , \end{array}$$

where the integrals over $\left[r_{1},r_{4}\right]$ are evaluated by the trapezoidal method with the interval $\left[r_{1},r_{4}\right]$ partitioned into the three sub-intervals $\left[r_{1},r_{2}\right]\cup \left[r_{2},r_{3}\right]\cup \left[r_{3},r_{4}\right]$, i.e, with the points at $r_{1}$, $r_{2}$, $r_{3}$, and $r_{4}$. Equation (24) will contribute to one row of $A_{i}$ and one row of $b_{i}$ as in Theorem 1. With the assumption that $A_{i}$ has full rank with ‘stride’ set to 3, by setting ‘stride’ to 3, the number of equations in the minimization problem in Algorithm 2 is two thirds less than that with ‘stride’ set to 1, and as a result, the computation load is reduced in the numerical minimization. It is remarked that with ‘stride’ equal to 3, all the four points at $r_{1},\ldots ,r_{4}$ are used by the trapezoidal method to evaluate the integrals over the interval $\left[r_{1},r_{4}\right]$ in (24), producing a more precise value of integral than the one that would be obtained with the two end points at $r_{1}$ and $r_{4}$ only. An example of calling adpSetModelFree is shown in Listing A4 in the Appendix A. Similarly, adpModelFree takes the output of adpSetModelFree as an argument to validate the options specified.

## 4. Applications to the Satellite Attitude Stabilizing Problem

In this section, we apply the ADPT to the satellite attitude stabilizing problem because a stabilization problem can be formulated as an optimal control problem. In the first example, the system model is known and the controller is computed by the function adpModelBased. The same problem is solved again in the second example by the function adpModelFree when the system dynamics is unknown. The source codes for these two examples are available at https://github.com/Everglow0214/The_Adaptive_Dynamic_Programming_Toolbox (accessed on 10 August 2021), where more applications of the toolbox can be found.

### 4.1. Model-Based Case

Let H denote the set of quaternions and $S^{3}=\left\{q\in H|∥q∥=1\right\}$. The equations of motion of the continuous-time fully-actuated satellite system are given by

(25) $$\begin{array}{cc} \dot{q} & =\frac{1}{2}q\Omega , \end{array}$$

(26) $$\begin{array}{cc} \dot{\Omega } & =I^{-1}\left(\left(I\Omega \right)\times \Omega \right)+I^{-1}u, \end{array}$$

where $q\in S^{3}$ represents the attitude of the satellite, $\Omega \in R^{3}$ is the body angular velocity vector, $I\in R^{3\times 3}$ is the moment of inertia matrix and $u\in R^{3}$ is the control input. The quaternion multiplication is carried out for qΩ on the right-hand side of (25) where Ω is treated as a pure quaternion. By the stable embedding technique [22], the system (25) and (26) defined on $S^{3}\times R^{3}$ is extended to the Euclidean space $H\times R^{3}$ [23,24] as

(27) $$\begin{array}{cc} \dot{q} & =\frac{1}{2}q\Omega -{\alpha (|q|}^{2}-1)q, \end{array}$$

(28) $$\begin{array}{cc} \dot{\Omega } & =I^{-1}\left(\left(I\Omega \right)\times \Omega \right)+I^{-1}u, \end{array}$$

where q∈H, $\Omega \in R^{3}$ and α>0.

Consider the problem of stabilizing the system (27) and (28) at the equilibrium point $\left(q_{e},\Omega_{e}\right)=\left(\left(1,0,0,0\right),\left(0,0,0\right)\right)$. The error dynamics is given by

$$\begin{array}{cc} {\dot{e}}_{q} & =\frac{1}{2}\left(e_{q}+q_{e}\right)e_{\Omega }-\alpha (|e_{q}+q_{e}{|}^{2}-1)\left(e_{q}+q_{e}\right),\\ {\dot{e}}_{\Omega } & =I^{-1}\left(\left(Ie_{\Omega }\right)\times e_{\Omega }\right)+I^{-1}u, \end{array}$$

where $e_{q}=q-q_{e}$ and $e_{\Omega }=\Omega -\Omega_{e}$ are state errors. Since the problem of designing a stabilizing controller can be solved by designing an optimal controller, we pose an optimal control problem with the cost integral (2) with $q\left(x\right)=x^{T}Qx$, where $x=\left(e_{q},e_{\Omega }\right)\in R^{7}$ and $Q=2I_{7\times 7}$, and $R=I_{3\times 3}$. The inertia matrix I is set to I=diag(0.1029,0.1263,0.0292). The parameter α that appears in the above error dynamics is set to α=1.

We set the option ‘xInit’ with three different initial states. For each initial state, the option ‘tSpan’ is set to [0,15]. We use the option ‘explSymb’ to set exploration signals; refer, for the usage of the option ‘explSysb’, to the user manual available at https://github.com/Everglow0214/The_Adaptive_Dynamic_Programming_Toolbox (accessed on 10 August 2021). For the initial control $u_{0}$, the default initial control is used, which is an LQR controller computed for the linearization of the error dynamics around the origin with the weight matrices $Q=2I_{7\times 7}$ and $R=I_{3\times 3}$. We then call the function, adpModelBased, to generate controllers of degree d=1,2,3. The computation time taken by the function, adpModelBased, to produce the controllers are recorded in Table 1. For the purpose of comparison, we also apply Al’brekht’s method with the non-linear systems toolbox (NST) [16] to produce controllers of degree d=1,2,3 for the same optimal control problem, and record their respective computation time in Table 1. For comparison in terms of optimality, we apply the controllers to the system (27) and (28) for the initial error state $x_{0}=\left(\left(\cos\left(\theta /2\right)-1,\sin\left(\theta /2\right),0,0\right),\left(0,0,0\right)\right)$ with θ=1.99999π and compute their corresponding values of the cost integral in Table 1. Since we do not know the exact optimal value of the cost integral $J(x_{0},u)$ for this initial state, we employ the software package called ACADO [18] to numerically produce the optimal control for this optimal control problem with the given initial state. We note that both NST and ACADO are model-based.

We can see in Table 1 that ADPT in the model-based mode is superior to NST in terms of optimality, and ADPT (model-based) for d=2,3 is on par with ACADO in terms of optimality. Notice however that ACADO produces an *open-loop* optimal control for each given initial state, which is a drawback of ACADO, while ADPT produces a *feedback* optimal control that is independent of initial states. Moreover, even for the given initial state ACADO takes a tremendous amount of time to compute the open-loop optimal controller. From these observations, we can say that ADPT in the model-based mode is superior to NST and ACADO in terms of optimality, speed, and usefulness all taken into account.

### 4.2. Model-Free Case

Consider solving the same optimal problem as in Section 4.1, but the system dynamics in (25) and (26), or equivalently the error dynamics are not available. Since we do not have real trajectory data available, for the purpose of demonstration we generate some trajectories with four initial states for the error dynamics, where the same initial control $u_{0}$ and exploration signals η are used as the model-based case in Section 4.1. The simulation for data collection is run over the time interval [0,20] with the recording period being 0.002 s, producing 10,000 = 20/0.002 sampled points for each run. For the function adpModelFree, the option of ‘stride’ is set to 4. Then, the function, adpModelFree, is called to generate controllers of degree d=1,2,3, the computation time taken for each of which is recorded in Table 1. For the purpose of comparison in terms of optimality, we apply the controllers generated by adpModelFree to the system (27) and (28) with the initial error state $x_{0}=\left(\left(\cos\left(\theta /2\right)-1,\sin\left(\theta /2\right),0,0\right),\left(0,0,0\right)\right)$ with θ=1.99999π and compute the corresponding values of the cost integral; see Table 1 for the values.

From Table 1, we can see that ADPT in the model-free mode takes more computation time than ADPT in the model-based mode, and the cost integrals by ADPT in the model-free working mode is slightly higher than those in the model-based working mode, since the integrals in the iteration process are evaluated less accurately. However, ADPT in the model-free mode is superior to NST in terms of optimality and to ACADO in terms of computation time. More importantly, it is noticeable that the result by model-free ADPT is comparable to model-based ADPT, which shows the power of data-based adaptive dynamic programming and the ADP toolbox.

**Figure 1:**
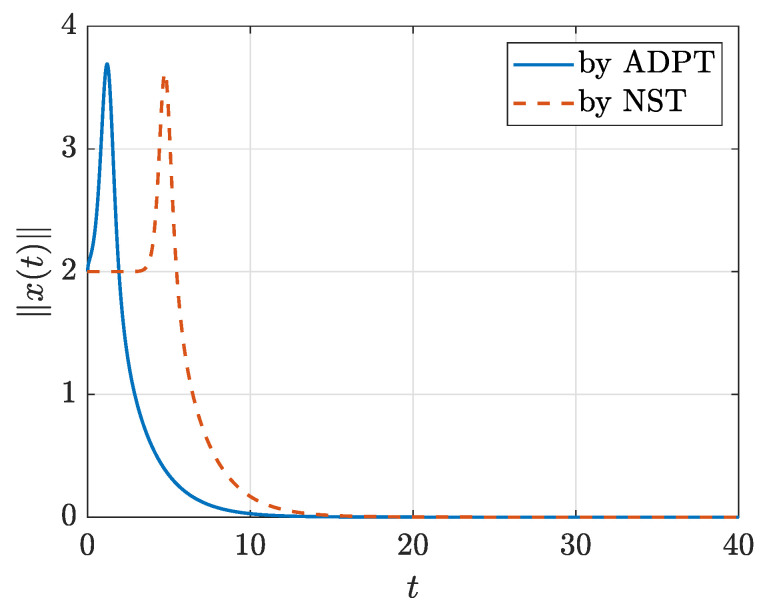
The state errors ∥x(t)∥ with the controllers of degree 3 generated by ADPT in the model-free working mode and by NST.

To see how the computed optimal controller works in terms of stabilization, the norm of the state error under the control with d=3 generated by ADPT in the model-free mode is plotted in Figure 1 together with the norm of state error by the NST controller with degree 3. We can see that the convergence to the origin is faster with the model-free ADP controller than with the controller by NST that is model-based. This comparison result is consistent with the comparison of the two in terms of optimality.

### 4.3. Discussion

To compare with other toolboxes on ADP or RL, we investigate MATLAB reinforcement learning toolbox with the same control problem. Equations (27) and (28) are discretized using the 4th order Runge–Kutta method to construct the environment in reinforcement learning toolbox. The integrand in (2) is taken as the reward function. The deep deterministic policy gradient (DDPG) algorithm [25] is selected to train the RL agent since the control input in (26) is continuous. However, it is found in simulations that the parameters of the agent generally diverge even after a long training time and the system cannot be stabilized. The reason probably is that by setting only parameters of the exploration signal of standard normal distribution such as mean and deviation rather than choosing an exploration signal of a specific form, the system states may go to infinity in some episodes. Although one may stop the episode before all steps run out in such a situation, the experiences saved in the replay buffer may be detrimental to the training. On the other hand, the options provided by ADPT allow the user to determine what kind of trajectories to be used so that the optimal feedback control may be found quickly.

## 5. Conclusions and Future Work

The adaptive dynamic programming toolbox, a MATLAB-based package for optimal control for continuous-time control-affine systems, has been presented. By employing the adaptive dynamic programming technique, we propose a computational methodology to approximately produce the optimal control and the optimal cost function, where the Kronecker product used in previous literature is replaced by Euclidean inner product for less memory consumption at runtime. The ADPT can work in the model-based mode or in the model-free mode. The model-based mode deals with the situation where the system model is given while the model-free mode handles the situation where the system dynamics are unknown but only system trajectory data are available. Multiple options are provided, such that the ADPT can be easily customized. The optimality, the running speed, and the utility of the ADPT are illustrated with a satellite attitude stabilizing problem.

Currently control policies and cost functions are approximated by polynomials in the ADPT. As mathematical principles of neural networks are being revealed [26,27], we plan to use deep neural networks in addition to polynomials in the ADPT to approximately represent optimal controls and optimal cost functions to provide users of the ADPT more options.

## Figures and Tables

**Table 1:** Costs at $x_{0}$ and computation time by ADPT, NST, and ACADO. $J(x_{0},u)$ denotes the integral cost of the corresponding control *u* for initial state $x_{0}$. ‘Time [s]’ denotes the computation time taken by the method to obtain the controller.

		$J(x_{0},u)$	Time [s]
ADPT (model-based)	d=1	37.8259	1.5994
	d=2	33.6035	3.2586
	d=3	33.4986	13.1021
ADPT (model-free)	d=1	43.8308	0.9707
	d=2	36.8319	3.3120
	d=3	37.4111	64.8562
NST	d=1	208.9259	0.2702
	d=2	94.6868	0.6211
	d=3	64.0721	3.6201
ACADO	-	32.6000	2359.67

## Appendix A

**Listing A1.** An example of the model-based mode.

1	n = 2; *%* *state* *dimension*
2	m = 1; *%* *control* *dimension*
3	*%%* *Symbolic* *variables*.
4	syms x [n,1] real
5	syms u [m,1] real
6	syms t real
7	
8	*%%* *Define* *the* *system*.
9	k1 = 3; k2 = 2; k3 = 2; k4 = 5;
10	f = [x2;
11	(-k1*x1-k2*x1^3-k3*x2)/k4];
12	g = [0;
13	1/k4];
14	
15	*%%* *Define* *the* *cost* *function*.
16	q = 5*x1^2 + 3*x2^2;
17	R = 2;
18	
19	*%%* *Execute* *ADP* *iterations*.
20	d = 3; *%* *approximation* *degree*
21	[w,c] = adpModelBased(f,g,x,n,u,m,q,R,t,d);

**Listing A2.** An example of the model-free mode.

1	n = 2; *%* *state* *dimension*
2	m = 1; *%* *control* *dimension*
3	
4	*%%* *Define* *the* *cost* *function*.
5	q = @(x) 5*x(1)^2 + 3*x(2)^2;
6	R = 2;
7	
8	*%%* *Generate* *data*.
9	syms x [n,1] real
10	syms t real
11	k1 = 3; k2 = 2; k3 = 2; k4 = 5;
12	*%* *System* *dynamics*.
13	f = [x2;
14	(-k1*x1-k2*x1^3-k3*x2)/k4];
15	g = [0;
16	1/k4];
17	
18	F = [1, 1] *%* *feedback* *gain*
19	
20	*%* *Exploration* *signal*.
21	eta = 0.8*(sin(7*t)+sin(1.1*t)+sin(sqrt(3)*t)+...
22	sin(sqrt(6)*t));
23	e = matlabFunction(eta,’Vars’,t);
24	
25	*%* *To* *be* *used* *in* *the* *function* *ode45*.
26	dx = matlabFunction(f+g*(-F*x+eta),’Vars’,{t,x});
27	
28	xInit = [-3, 2;
29	2.2, 3];
30	tSpan = [0:0.002:6;
31	0:0.002:6];
32	odeOpts = odeSet(’RelTol’,1e-6,’AbsTol’,1e-6);
33	
34	t_save = [];
35	x_save = [];
36	for i = 1:size(xInit,1)
37	[time, states] = ode45(@(t,x)dx(t,x),tSpan(i,:),...
38	xInit(i,:),odeOpts);
39	t_save = [t_save; time];
40	x_save = [x_save; states];
41	end
42	
43	u0_save = -x_save * F;
44	eta_save = e(t_save);
45	
46	*%%* *Execute* *ADP* *iterations*.
47	d = 3; % approximation degree
48	[w,c] = adpModelFree(t_save,x_save,n,u0_save,m,...
49	eta_save,d,q,R);

**Listing A3.** A demonstration of calling the function adpSetModelBased.

1	*%% The user may specify settings*.
2	xInit = [-3, 2;
3	2.2, 3];
4	tSpan = [0, 10;
5	0, 8];
6	
7	syms t real
8	eta = [0.8*sin(7*t)+sin(3*t);
9	sin(1.1*t)+sin(pi*t)];
10	
11	adpOpt = adpSetModelBased(’xInit’,xInit,’tSpan’,tSpan,...
12	’explSymb’,eta);
13	
14	*%% Execute ADP iterations*.
15	[w,c] = adpModelBased(f,g,x,n,u,m,q,R,t,d,adpOpt);

**Listing A4.** A demonstration of calling the function adpSetModelFree.

1 *%%* *The* *user* *may* *specify* *settings*.
2 adpOpt = adpSetModelFree(’stride’,2);
3
4 *%%* *Execute* *ADP* *iterations*.
5 [w,c] = adpModelFree(t_save,x_save,n,u0_save,m,...
6 eta_save,d,q,R,adpOpt);
